# Editorial: Talking and Cure – What's Really Going On in Psychotherapy

**DOI:** 10.3389/fpsyg.2021.693300

**Published:** 2021-09-09

**Authors:** Anssi Peräkylä, Michael B. Buchholz

**Affiliations:** ^1^University of Helsinki, Helsinki, Finland; ^2^International Psychoanalytic University Berlin, Berlin, Germany

**Keywords:** psychotherapy, conversation analysis, psychotherapy process, social interaction, social science, linguistics

Nearly 130 years ago Bertha Pappenheim coined the term “talking cure” while she was in hypnotic treatment with Dr. Joseph Breuer in Vienna. Freud adopted this term and it was echoed through a century: “…it all started with the talking cure” (Kächele, 1992, p. 2). Even though talk is so central in psychotherapy, up until now, we know relatively little about the actual structure and properties of it.

Interactional details of psychotherapy have been investigated by anthropologists and linguists since 1960s (e.g., Pittenger et al., 1960; Scheflen, 1973). In the clinical world, the most important pioneers were Horst Kächele and Helmut Thomä, who in 1970s started a large textbank of psychotherapy transcripts that has been used ever since in quantitative and qualitative studies (Mergenthaler and Kächele, 1988) as well as a resource for highly influential textbooks (Thomä and Kächele, 1985/2021, 1994). In quantitative, clinically oriented research, counting of words, topics of talk or length of pauses has revealed patients' style of talk or underlying conflictual social relations (e.g., Luborsky and Crits-Christoph, 1988). Some researchers, however, considered such quantitative approach too simple, insensitive to the details of expression and action which centrally contribute to the therapeutic character of the talk. Gradually, the way for paying more attention to the details of a therapeutic conversation was opened, as the cooperation between psychotherapy researchers and conversation analysts was begun some 20 years ago (see Peräkylä et al., 2008).

This Research Topic presents some of the developments of the conversation analytical (CA) line of research. The 11 papers touch upon four largely overlapping themes.

(1) **Alignment and resistance**. Alignment means participants' collaboration in maintaining actions and activities in therapy, while resistance—in CA terms—means that one participant does not go along in the course of action initiated by the other. Muntigl et al. examined a particular therapeutic technique called chair work: the clients' ways of ways of resisting the therapists' proposals of such work, and the therapists' ways of resolving the client resistance. These ways include proffering of alternatives, as well as accounting for and elaborating on the proposals, and they are intertwined with negotiation of deontic and epistemic relations between the therapist and the client. Scarvaglieri takes up the potential tension between the building of a positive client-therapist relation on one hand, and the therapist's disalingment with the client's communicative activities on the other hand. Examining first encounters in therapy, he shows that such disalingment is necessary for the achievement of the interactive and institutional goals in therapy. Buchholz et al. investigate alignment and resistance during the first 20 min of a therapy session with a 4-year old traumatized child. What they call “doing contrariness” involves the child's practices producing epistemic and affiliative disruptions. The paper also shows the therapists' strategies for preserving or restoring the affiliative dimension of the relationship. Janusz et al. take up couple therapy with clients who are diagnosed with narcissistic personality disorder. They show how the narcissistic clients work to ensure their control of the unfolding of the interaction, by not answering the therapist's questions, by blocking the development of the conversational topic, and by conspicuous displays of their interactional independence.

(2) **Organization of affect**. Emotions and affects are at the heart of psychotherapy, and CA offers a way to describe the interactional display, expression and regulation of them in a very close distance. Guxholli et al. investigate the therapists' ways of managing a prolonged disagreement with the patient. They show particular interactive trajectories where the therapist, in the midst of such disagreement, briefly affiliates with the patient by producing a collaborative conversational move, only to return to the disagreement thereafter. The local affiliation is thus in the service of a prolonged disagreement. Muntigl explores talk about the client's upsetting experiences in a single session of client-centered therapy. He shows the therapist's ways of focusing on the client's distress, and the client's ways of opposing this. Through repeated episodes the client's display of distress and the therapist's responses, the participants eventually secure extended emotional work. Avdi and Evans bring together three analytic resources on management of affect. Employing CA, they investigate the client's narration about her anger and guilt and the therapist's responses to it; with psychoanalytic concepts, they explore the possible conflicts and unconscious processes pertaining to these interactions; and by measuring the autonomic nervous system responses, they examine the participants' physiological arousal during the narration and formulations.

(3) **Specific linguistic and non-linguistic resources**. Psychotherapeutic talk—as any spoken interaction—rests upon the participants' command of numerous lexical, grammatical, prosodic and kinetic resources, by means of which they produce and recognize actions that can have therapeutic functions. Etelämäki et al. investigate the therapists' use of two forms of person reference in Finnish language in responses to the clients' complaints. The zero-person (a form that lacks grammatical subject) is used in affiliating responses, whereas the second person (addressing the client directly) is used for reconstructing the client's past history. Knoll et al. examine how silences that occurs after the therapist's continuer receive their meaning in and through the participants' next actions. In most cases, the silence is followed by therapist's turn where they shift the topic, or by the client's turn where they continue on topic. Only in some cases, the therapist, in their next turn, formulates the meaning of silence as a therapeutic event.

(4) **Specific interactional trajectories**. Talk in psychotherapy is distinguishable from talk in many other settings. The distinctness of psychotherapy rests, in part, on the particular, “psychotherapy specific” action sequences. Deppermann et al. show that in psychotherapeutic interaction, the therapists sometimes respond to the clients' narratives, not by taking up the semantic content of the narration, but by topicalizing the “performative self” that the patient enacts through the narration. By doing so, the therapists also focus away from, and even challenge, the identity claims that the content of the narration conveyed. Ekberg investigated sequences where the therapist proposes connections between two experiences that have been discussed separately—for example tying what is currently being talked about, with something that the client told in a prior session. Such connections can contribute to the psychological account of the client's experience.

CA in psychotherapy shows the complexity of the therapeutic task in much more details than could be observed by therapy theories alone (Buchholz and Kächele, 2017). By looking binocularly, clinically and conversationally, onto what is going on in the treatment room, we see the processes of balancing therapeutic alliance and affectivity, alignment and resistance, institutional frames and individualized projects and others. We hope that this Research Topic will show how CA of psychotherapy is of value for linguists, social scientists, psychotherapist and psychotherapy trainers alike.

## Author Contributions

MBB and AP planned and wrote the article collaboratively.

## Conflict of Interest

The authors declare that the research was conducted in the absence of any commercial or financial relationships that could be construed as a potential conflict of interest.

## Publisher's Note

All claims expressed in this article are solely those of the authors and do not necessarily represent those of their affiliated organizations, or those of the publisher, the editors and the reviewers. Any product that may be evaluated in this article, or claim that may be made by its manufacturer, is not guaranteed or endorsed by the publisher.
